# Comparative genomics of *Leptospira santarosai* reveals genomic adaptations in bovine genital strains

**DOI:** 10.3389/fmicb.2024.1517151

**Published:** 2025-01-07

**Authors:** Maria Isabel Nogueira Di Azevedo, Frederico Kremer, Camila Ezepha, João Pedro Gomes Greco, Isadora Cosenza Vieira da Silva, Pascale Bourhy, Walter Lilenbaum

**Affiliations:** ^1^Laboratory of Veterinary Bacteriology, Biomedical Institute, Fluminense Federal University, Niterói, Rio de Janeiro, Brazil; ^2^Laboratory of Bioinformatics - Omixlab, Technological Development Center, Federal University of Pelotas, Capão do Leão, Rio Grande do Sul, Brazil; ^3^Institut Pasteur, Biology of Spirochetes Unit, National Reference Center for Leptospirosis, Paris, France

**Keywords:** leptospirosis, BGL, veterinary microbiology, WGS, infectious disease

## Abstract

Bovine genital leptospirosis (BGL) is a silent and chronic reproductive syndrome associated with reproductive failures that result in animal suffering and substantial financial losses for farmers. Important aspects of the interactions between the host and the pathogen during chronic leptospirosis have been well described in the kidney, but little is known about the genital infection mechanisms. The present study sheds light on the pathophysiology of BGL based on comparative genomic analysis of renal versus genital isolates of *Leptospira santarosai* genomes, an endemic species on Latin America. A significant number of genes were exclusive of the genital strains, with emphasis on genes associated with cell wall/membrane/envelope biogenesis, mobilome: prophages and transposons, and signal transduction mechanisms. Overall, these gene clusters play crucial roles in bacterial colonization and evasion of the immune response, which can reflect leptospiral tissue tropism to the genital niche. We provide new insights into the pathophysiology of an important and neglected syndrome in bovine, helping to elucidate the evolution of adaptation of leptospires in the genital tract of cows.

## 1 Introduction

*Leptospira* sp. is a highly diverse spirochete bacteria that comprises several taxa based on a genetic classification subdivided into 64 genomic species, including saprophytic, intermediate and pathogenic species. Only eight pathogenic species are responsible for human and animal infections: *L. interrogans, L. kirschneri, L. noguchi, L. santarosai, L. mayottensis, L. borgpetersenii, L. alexanderi,* and *L. weilli* (Vincent et al., 2019). This group includes the etiologic agents of the globally neglected zoonotic disease leptospirosis (Picardeau, 2020). Serological classification is important for epidemiological purposes. It defines serovars based on the lipopolysaccharides of the outer membrane, and, according to its homogeneity, groups the serovars into serogroups. Using this approach, approximately 24 serogroups containing over 250 serovars have thus far been recognized (Hagedoorn et al., 2024). Notably, host-serogroup adaptability has been demonstrated and associated with asymptomatic and or chronic/silent disease (Xu et al., 2016). The two classifications are independent and their integrative use is recommended.

In bovine, leptospirosis is one of the major causes of reproductive failures and is associated with abortions, estrus repetition, stillbirths, the birth of weak calves, a decrease in growth parameters, and a drop in milk production (Fávero et al., 2017), all factors that result in substantial financial losses for farmers. This silent and chronic reproductive syndrome is formally called bovine genital leptospirosis (BGL). Currently, strains from the Sejroe serogroup are recognized as the main agents of BGL due to the syndrome’s adaptability to the bovine host (Loureiro and Lilenbaum, 2020).

Within the Sejroe serogroup, the main serovars are Hardjoprajitno (*L. interrogans*) and Hardjobovis (*L. borgpetersenii*) yet the serovar Guaricura (*L. santarosai*) has also been frequently identified in cattle, especially in Latin America (Kremer et al., 2015; Loureiro et al., 2016; Aymée et al., 2022b; Chinchilla et al., 2023) and its significance as a BGL agent has grown.

Important aspects of the interactions between the host and the pathogen during chronic leptospirosis have been well described in the kidney (Monahan et al., 2009). For years, studies have focused on urine/renal tissues, with kidneys being considered an immune-privileged and primary site of infection that facilitates persistent colonization by leptospires. Leptospiral infection in the reproductive tract has traditionally been considered secondary to renal pathogenesis. However, recent studies involving reproductive tract samples have demonstrated the genital tract as an important extra-renal site of infection by *Leptospira* spp. The spirochaete has been identified on the molecular level in the uterus (Cabral Pires et al., 2018; Di Azevedo et al., 2020; Aymée et al., 2021, 2022a, 2023; Borges et al., 2024), vaginal fluid (Loureiro et al., 2016; Aymée et al., 2022b), follicular fluid (Di Azevedo et al., 2021; Dos Santos Pereira et al., 2022), and cervicovaginal mucus (Aymée et al., 2021, 2022a, 2023; Borges et al., 2024). Most of the cows studied had a history of reproductive problems, including abortion, chronic subfertility, and embryonic mortality.

Although *Leptospira* spp. infections in the urinary and genital tract have a distinct pathophysiology with different clinical manifestations, the dynamics of genital infection, adaptability to the uterine environment, and mechanisms of pathogenesis are not fully understood. The use of Whole Genome Sequencing (WGS) has helped to further elucidate the evolution of the virulence of the pathogenic species of *Leptospira* associated with the urinary tract in several hosts (Xu et al., 2016; Santos et al., 2018; Koizumi et al., 2020; Ramli et al., 2021; de Oliveira et al., 2022). However, studies applying WGS to genital *Leptospira* strains have never been conducted. Recently, our group provided the first draft genomes of three *L. santarosai* strains isolated from the vaginal fluid of Brazilian cows with reproductive disorders (Di Azevedo et al., 2023). As urinary *L. santarosai* genomes are also available (Kremer et al., 2015; Di Azevedo et al., 2023), the present study aims to shed light on the pathophysiology of BGL using comparative genomic analyses of renal versus genital isolates.

## 2 Materials and methods

### 2.1 Samples, culturing, and initial genetic characterization of strains

The *Leptospira santarosai* strains used in the present study included urinary (2014_U76, 2013_U160, 2013_U164, and 2013_U233) and genital isolates (2013_VF52, 2014_VF66, and VF237), all associated with bovine genital leptospirosis. They were characterized using the serology and *sec*Y gene sequencing conducted by our group in previous studies on cows from the State of Rio de Janeiro, Brazil (Hamond et al., 2015; Loureiro et al., 2016; Aymée et al., 2022b). Information regarding the strains included in the present study is summarized in Table 1.

**Table 1:** Summarized information regarding *Leptospira santarosai* strains isolated from bovine included in the present study.

Strain	Original references	Biological source	Serogroup	Genome references	Acession number
2013_VF52	Loureiro et al. (2016)	Vaginal fluid	Sejroe	Di Azevedo et al. (2023)	JAUOTG000000000
2014_VF66	Loureiro et al. (2016)	Vaginal fluid	Sejroe	Di Azevedo et al. (2023)	JAUOTF000000000
2014_U76	Loureiro et al. (2016)	Urine	Sejroe	Di Azevedo et al. (2023)	JAUOTE000000000
VF237	Aymée et al. (2022a)	Vaginal fluid	Sejroe	Di Azevedo et al. (2023)	JAUOTH000000000
2013_U160	Hamond et al. (2015)	Urine	Sarmin	Kremer et al. (2015)	LAYP00000000
2013_U164	Hamond et al. (2015)	Urine	Tarassovi	Kremer et al. (2015)	LAZM00000000
2013_U233	Hamond et al. (2015)	Urine	Grippotyphosa	Kremer et al. (2015)	LAZN00000000

Briefly, after collection, the samples were immediately seeded into tubes with EMJH medium supplemented with the antimicrobial cocktail STAFF (Loureiro et al., 2015). The tubes were kept at room temperature and transported to the laboratory. All cultures were maintained at 29°C and examined weekly for 16 weeks using dark field microscopy. As soon as pure cultures were obtained, with few passages (average 4), they were frozen at liquid nitrogen (−196°C) in the Collection of Bacterial Cultures of Veterinary Interest (Federal Fluminense University, Brazil). Cultures were thawed exclusively further for the analyses of the present study.

The isolates were tested by Microscopic Agglutination Test (MAT) against a panel of rabbit antisera of 32 reference serovars representing 24 serogroups [provided by KIT (formerly the Royal Tropical Institute), Amsterdam], as recommended (Haake and Levett, 2015). DNA was extracted using the Wizard SV Genomic DNA Purification System^®^ (Promega, Madison, USA) according to the manufacturer’s instructions. Genotyping was performed using the *sec*Y locus as described by Ahmed et al. (2006). PCR products were directly sequenced in both directions using the Big Dye Terminator 3.1 Cycle Sequencing Kit (Life Technologies, Foster City, USA) on an ABI 3730XL Genetic Analyzer (Life Technologies, Carlsbad, CA, USA) on the PDTIS/Fiocruz Bioinformatics Platform RPT01A. After Pairwise/Blast/NCBI comparisons were performed, the sequences were taxonomically identified as *L. santarosai* (>98%).

### 2.2 Genome sequencing, assembly, and annotation

Whole genome sequences from the strains 2013_U160, 2013_U164, and 2013_U233 were previously sequenced by Kremer et al. (2015), as were those from the strains 2014_U76, 2013_VF52, 2014_VF66, and VF237, which were sequenced by Di Azevedo et al. (2023) (Table 1). Briefly, based on the high-quality genomic DNA obtained from 5 × 10^10^ to 2 × 10^11^ pelleted *L. santarosai* cultures, WGS was performed on Illumina’s (San Diego, California, USA) MiSeq Sequencing System applying a 150 × 2-bp run and starting with a genomic library prepared using Illumina DNA Prep (previously known as Nextera DNA Flex). The quality of the reads was evaluated using fastQC, and the genomes were assembled *de novo* using the Unicycler 0.4.8 (Wick et al., 2017) workflow under default parameters. The annotation was added using the NCBI Prokaryotic Genome Annotation Pipeline. Additionally, for comparative analyses involving the pangenome of *L. santarosai*, a reference sequence of human origin, the *L. santarosai* serovar Shermani strain LT 821, was also included, as it is a pioneer strain already well characterized, being useful for visualizing the distribution and location of genes and for comparative analyses purposes (Chou et al., 2014).

As *L. santarosai* str. 2013_FV52 is a pioneer strain in several other studies (Loureiro et al., 2016; Martins et al., 2022; Barbosa et al., 2023), it was chosen for the circular representation of the genome, with predicted Coding DNA Sequences (CDSs). For this purpose, its draft genome (GenBank: JAUOTE010000000) was aligned and ordered based on the genome of the *L. santarosai* serovar Shermani str. LT 821 (GenBank: NZ_CP006694.1, NZ_CP006695.1) using CONTIGuator (Galardini et al., 2011), and the circular genome plot was produced using the program DNAPlotter (Carver et al., 2009) from the Artemis package (Berriman and Rutherford, 2003).

### 2.3 Comparative genomic analysis

Genomes were obtained from NCBI using the NCBI Datasets service.^1^ They were retrieved in GenBank File Format (GBFF) and converted to GFF3 files using BioPerl.^2^ The GFF3 files for all genomes were then analyzed using Roary (Page et al., 2015) under default settings to ascertain the pangenome and core-genome estimations. Roary operates by first clustering genes from all input genomes using a sequence identity threshold, typically set at 95%, to group similar genes together. This clustering is crucial for distinguishing between core and accessory genes. The software then generates a matrix of gene presence and absence, which is used to define the core genome as the set of genes present in all strains (Page et al., 2015).

A maximum-likelihood (ML) tree based on *L. santarosai* genomes was constructed using the Hasegawa-Kishino-Yano 85 model (HKY85) on PhyML 3.0 software (Guindon et al., 2010). Based on the Roary outputs, the core genomes of the urinary and genital strains were defined as the genes present in all members of each group. Functional annotations of the pangenome and each Cluster of Orthologous Groups (COG) category (Galperin et al., 2021) were performed using COGClassifier.^3^ A Fisher exact test followed by a Bonferroni correction of the *p*-values were performed to identify the differences in the distribution of COG categories in the genes conserved in each group of strains. All data analysis was performed using Python and the libraries BioPython,^4^ Pandas,^5^ SciPy,^6^ and Statsmodels.^7^ An additional functional annotation was performed using the InterProScan webserver.^8^

Additionally, to provide more informative results, we compared the identities of the genomes and genes identified in the strains of the present study with other *L. santarosai* genomes from other hosts. For this purpose, a BLAST search was performed using the NCBI-BLAST+ package to identify genes homologous to the urinary and genital strain core gene sets in the dataset of *L. santarosai* genomes obtained from the NCBI RefSeq. Homologous genes were identified based on the following criteria: an e-value threshold of 1e-10 (Cai et al., 2009), a minimum identity of 70%, and a minimum query coverage of 70%, cutoff points already widely used in the search for homology (Janaki et al., 2021; Martins et al., 2023). The percentages of genes in the genital and urinary gene sets were computed for each genome.

## 3 Results

### 3.1 Descriptive genomic analysis

**Figure 1:**
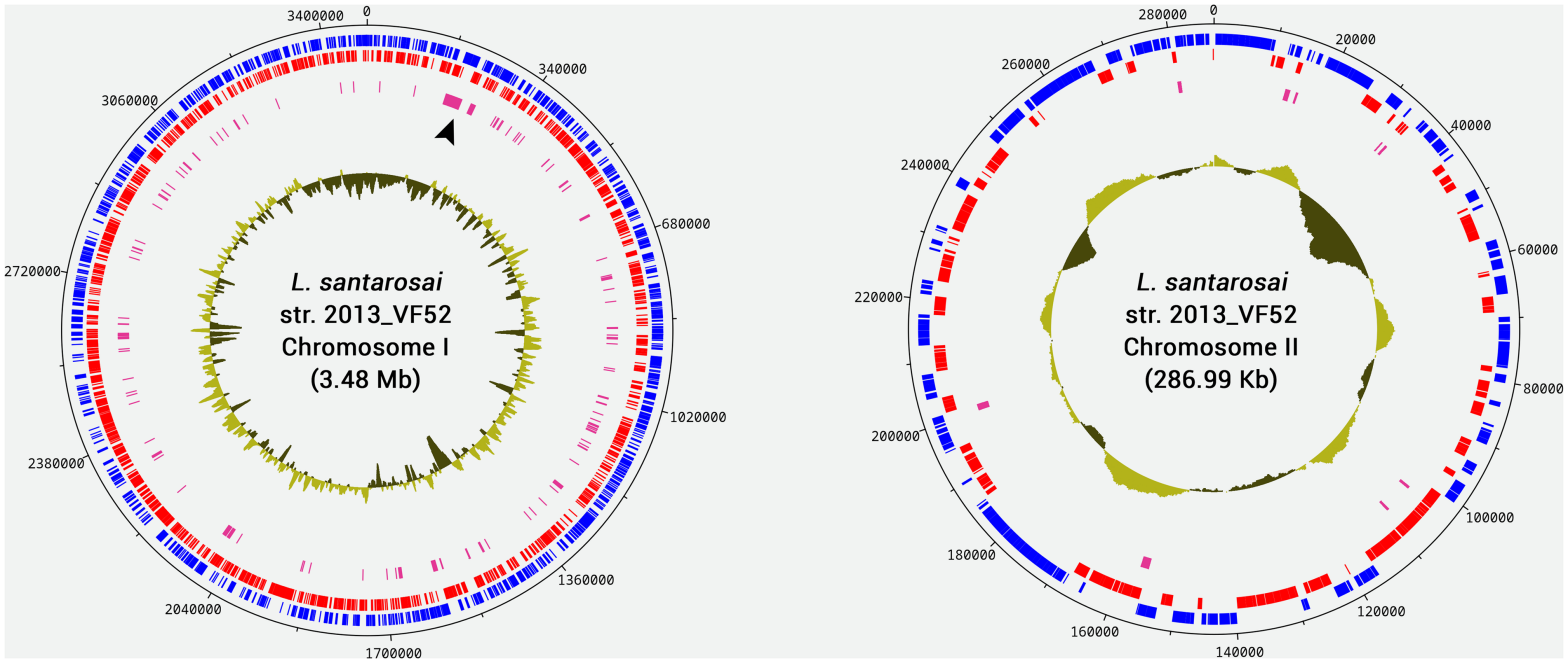
Circular genome diagram of the *Leptospira santarosai* strain 2013_VF52 genome generated based on chromosomes I and II of *L. santarosai* serovar Shermani str. LT 821 using CONTIGuator and DNAPlotter. The blue marks indicate the Coding DNA Sequences (CDSs) present in the forward strand, while the red marks indicate CDSs in the reverse strand. Pink marks indicate genes that are conserved exclusively in genital strains. The location indicated with the black arrowhead in chromosome I represents an extensive region highly conserved only in the genital strains. Deviations in the CG content are plotted inside each chromosome.

Based on the characteristics of the reference genome *L. santarosai* serovar Shermani strain LT 821, it was possible to infer that the genome of *L. santarosai* serovar Sejroe strain 2013_VF52 consists of two chromosomes, namely, the larger chromosome I (3.48 Mb, GC% 42.05) and the smaller chromosome II (286.99Kb, GC% 42.30) (Figure 1). It is possible to observe that chromosome 1 presents a higher number of genes conserved in the genital strains, with emphasis on a region between 17.000 and 34.000 bp, containing genes related to cell wall/membrane/envelope biogenesis. This structure was very similar to the *L. santarosai* strains obtained from vaginal fluid (2014_VF66 and VF237). Further details of the *Leptospira santarosai* genomes from bovine released to NCBI can be found in Kremer et al. (2015) and Di Azevedo et al. (2023).

### 3.2 Comparative genomic analysis

**Figure 2:**
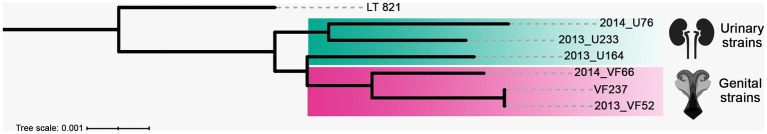
A maximum-likelihood (ML)-phylogenetic tree inferred from whole genome sequences of *L. santarosai* Sejroe serogroup strains of urinary (yellow rectangle) and genital (pink rectangle) origin. The *Leptospira santarosai* serovar Shermani strain LT 821 is the outgroup taxa.

An ML-phylogenetic tree created using the WGS of the *L. santarosai* strains revealed the polyphyletic origin of the urinary strains, while genital strains formed a monophyletic group separate from urinary strains (Figure 2). Importantly, strains VF237 and 2013_VF52 are identical.

**Figure 3:**
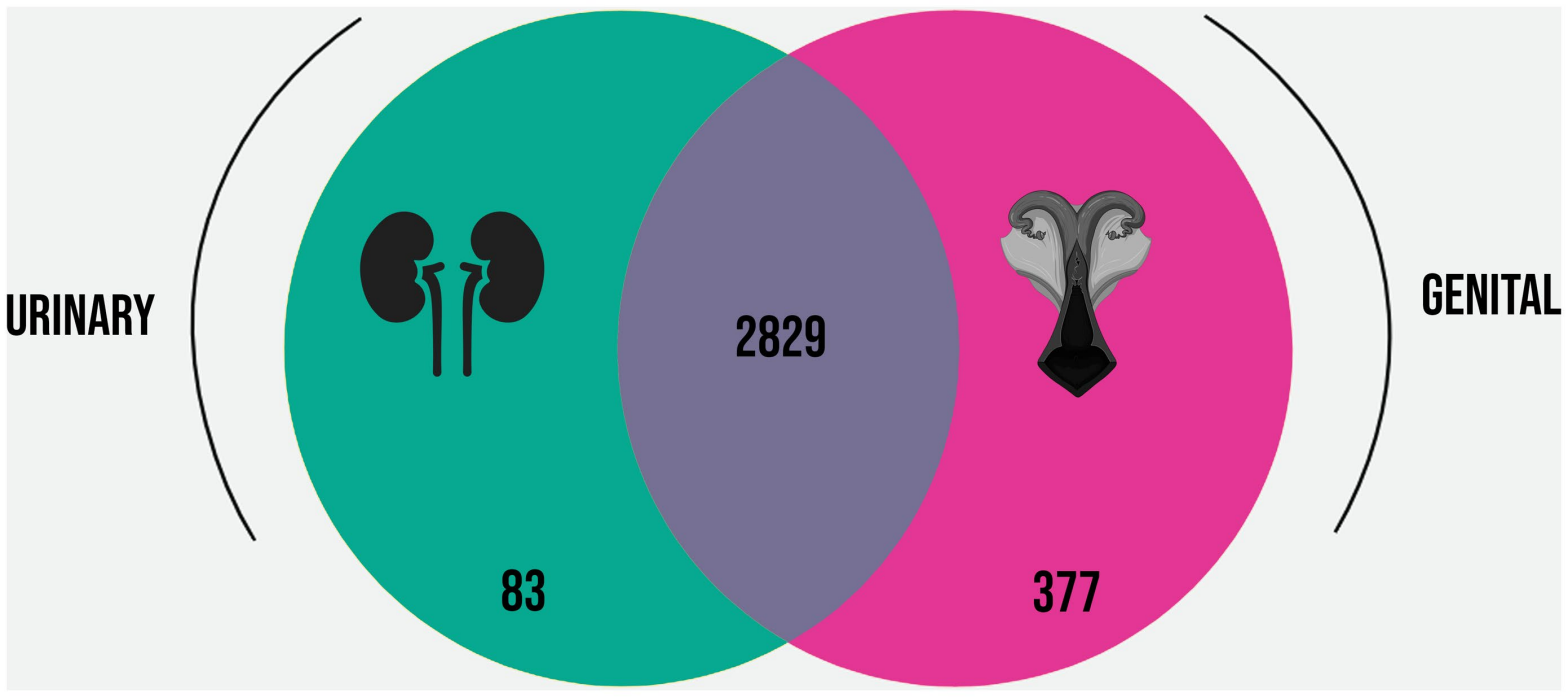
Venn diagram representing the overlap and differences in the core genomes of the *L. santarosai* strains derived from urinary (yellow) and genital (pink) samples.

Based on the Roary analysis, the pangenome of the selected strains was estimated in 4,969 genes, while the core genomes of the urinary and genital strains were estimated, respectively, in 2,912 and 3,206 genes. The differences between the core genomes are displayed in Figure 3, indicating that the 377 genes conserved in the genital strains were not conserved in the urinary strains, while 83 genes were conserved in the urinary strains but not in the genital strains. These subsets of genes were named the “urinary strain-specific” (USS) core genome and the “genital strain-specific” (GSS) core genome.

**Figure 4:**
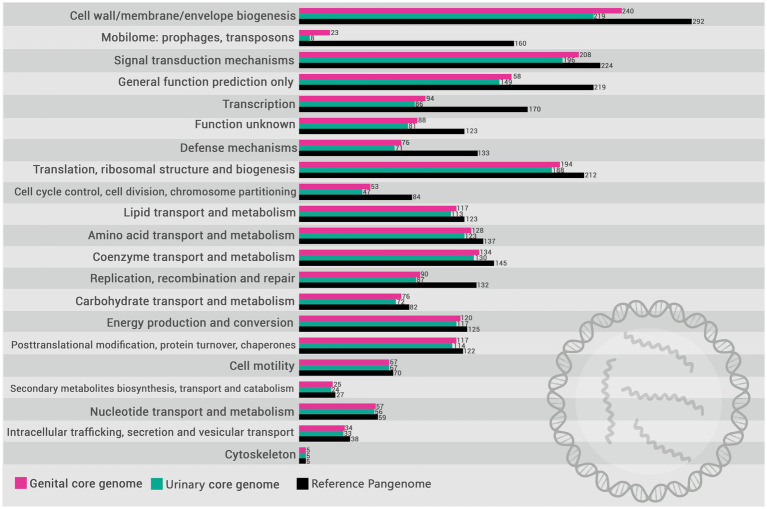
Distribution of COG (Cluster of Orthologous Groups) categories in the pangenome of *L. santarosai* (gray) and the core genomes of urinary (yellow) and genital strains (pink).

**Figure 5:**
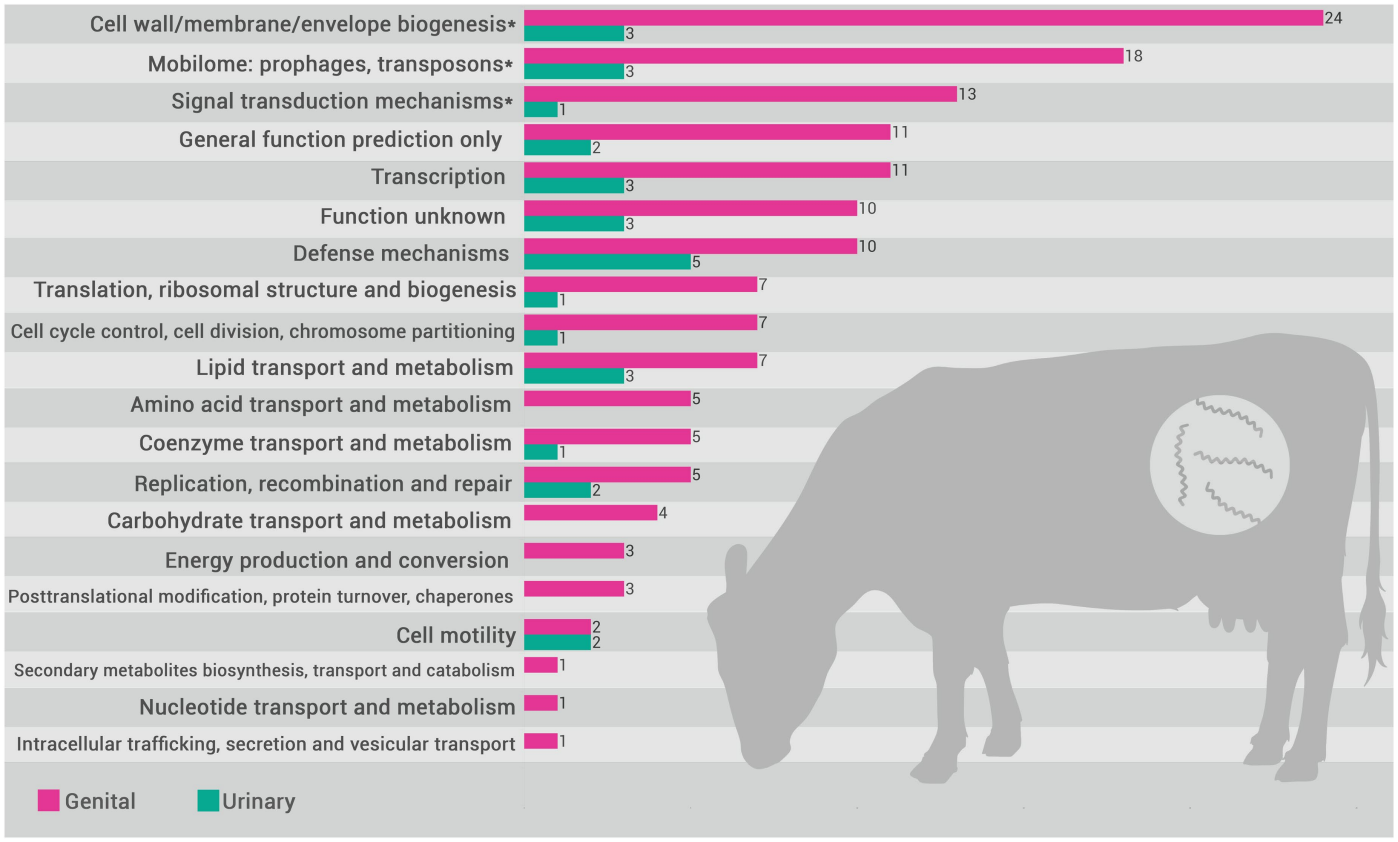
Distribution of COG (Cluster of Orthologous Groups) categories in *the L. santarosai* urinary strain specific (USS) (yellow) and genital strain specific (GSS) (pink) core genomes. Categories indicated with an asterisk (*) are those where a significant difference between categories (USS vs. GSS) was observed (*p* < 0.05).

Based on the functional annotation of the pangenome, the encoded proteins were mapped to COGs categories. The distribution of these categories in the pangenome of *L. santarosai* and the USS and GSS core genomes are illustrated in Figure 4. The functional enrichment analysis of the USS core genome as compared with the GSS core genome is shown in Figure 5. These results identify three COG categories as enriched in the GSS core genome (cell wall/membrane/envelope biogenesis, mobilome: prophages and transposons, and signal transduction mechanisms), with significant differences observed as regards the USS core genome (Supplementary material 1), indicating that proteins related to these biological processes are more present and/or conserved on genital strains. No enriched COG category was observed in the distribution of COG categories in the USS core genome analysis. The genes conserved in the GSS core genome and absent in the USS core genome relating to the aforementioned biological processes are presented in Supplementary material 2. Moreover, 24, 18, and 13 COGs were found to be related to cell wall/membrane/envelope biogenesis, mobilome, and signal transduction mechanisms, respectively, totaling 55 exclusive genital strain protein domains (Figure 5 and Supplementary material 2).

Regarding the percentages of genes in the USS and GSS gene sets as compared with the *L. santarosai* genomes obtained from the NCBI RefSeq, a maximum identity of 95% was found for urinary gene orthologues (*L. santarosai* strain M72/6–6 and serovar Grippotyphosa) and 71.9% for genital gene orthologues (*L. santarosai* strain M4/98 from serovar Guaricura) (Supplementary material 3).

## 4 Discussion

Although BGL is a well-recognized and characterized syndrome in many respects, the mechanisms involved in its pathophysiology in the genital tract and the adaptability profile of Sejroe strains to this site remain enigmatic. In this context, the results of the present study deliver new and important insights based on comparative genomics analyses of genital versus urinary strains. Notwithstanding that the strains are from the same host species (bovine) and geographic location (southeastern Brazil), phylogenetic analysis based on *L. santarosai* WGS revealed a monophyletic group including only genital strains (Figure 2) separate from urinary strains, suggesting important differences in the genome based on the biological origin of the leptospires. These evident genomic differences between genital and urinary strains may have important practical applications, such as optimizing diagnosis and developing an effective vaccine for BGL, based on the exploration of exclusive and conserved regions of genital strains. Importantly, it has been demonstrated that vaccination with bacterins cannot prevent colonization in the genital tract, and infected animals keep shedding leptospires (Martins et al., 2022), which can be explained by this high difference between urinary and genital strains, added to the peculiarities of the humoral response triggered in these two distinct sites.

Of note, a specific cluster including the strains 2013_VF52, 2013_VF66, and VF237 was observed in *sec*Y gene analysis (Aymée et al., 2022b; Aymée et al., 2022a), confirming the good taxonomic resolution of this genetic marker and its proper application for molecular epidemiological purposes, which is particularly useful for uncultured clinical samples when WGS cannot be performed. The strains 2013_VF52 and 2013_VF66 were first identified and characterized in asymptomatic cattle (Loureiro et al., 2016), suggesting the traditional “silent disease” associated with these strains. Importantly, it was demonstrated *in vivo* that the strains VF237 and 2013_VF52, which were demonstrated to be identical in the present study, determine chronic genital leptospirosis in the hamster model (Barbosa et al., 2023). Moreover, based on *sec*Y gene analysis, all strains were closely genetically associated with another strain from the Sejroe serogroup serovar Guaricura (UT27), which was isolated from a cow presenting embryonic loss, estrus repetition, and subfertility (Aymée et al., 2022b). This high genetic identity between strains is relevant in several scientific and practical perspectives, including: (i) the existence of a potential common source of infection; (ii) clonal expansion within bovine population; (iii) presence of exclusive characteristics that enhance its ability to cause chronic disease; (iv) supports the development of targeted strategies, such as vaccines; and (v) provides insights into the genetic stability of the strains and helps identify genetic variants linked to virulence or antimicrobial resistance.

Analysis of the urinary and genital *L. santarosai* core genomes revealed the presence of 377 genes exclusive of genital strains, a greater number than found exclusively in urinary strains (Figure 3). This may indicate the complexity of the genetic mechanisms involved in the colonization, maintenance, and survival of pathogenic leptospires from the Sejroe serogroup over long periods in the reproductive tract of cows. Additionally, when compared with another strain of bovine *L. santarosai* also from the Guaricura serovar but isolated from urine, the percentage of orthologous genital genes was only 71% (Supplementary material 3), indicating differences in gene constitution even within the same serovar.

Investigating the COGs (Figure 5 and Supplementary material 2), we identified that 55 GSS genes were significantly enriched compared with the USS genes, including three metabolic categories: cell wall/membrane/envelope biogenesis (*n* = 24), mobilome (*n* = 18), and signal transduction mechanisms (*n* = 13). Overall, these gene clusters play crucial roles in bacterial colonization and evasion of the immune response, and may contribute significantly to leptospiral tissue tropism and adaptation to the genital niche. It is unsurprising that genes related to cell wall/membrane/envelope biogenesis are significantly more prevalent in genital strains (*n* = 24 in GSS vs. *n* = 3 in USS). Cell wall biogenesis is essential for maintaining the structural and functional integrity of bacteria, allowing them to resist adverse environmental conditions, in addition to facilitating the colonization of specific niches in the host (Mayer and Dörr, 2019). Both kidney and genital infections caused by Sejroe serogroup strains are characterized by a host-pathogen biological equilibrium, with leptospires successfully evading the immune response, causing a silent and chronic disease (Loureiro and Lilenbaum, 2020). However, it is recognized that the kidney is an immune-privileged site that facilitates *Leptospira* colonization, with the absence of “complement one” a main hypothesis for how the leptospires evade the renal immune response (Monahan et al., 2009). Conversely, although the immunopathogenesis of the leptospiral infection in the genital tract remains unclear, it has been demonstrated that bovine cervicovaginal mucus contains high levels of immune-active proteins such as immunoglobulin A (IgA), lactoferrin, and lysozyme, which protect against infection by blocking adhesion and mediating microbial killing (Adnane et al., 2018). Therefore, the mechanisms of adhesion and maintenance of cell wall integrity for long periods in a hostile environment are essential for leptospiral survival. Similarly, COG analysis revealed that cell wall/membrane/envelope biogenesis genes were also differentially expressed in *Brucella abortus*, a bacterium also associated with reproductive failure in cattle (Sun et al., 2023).

Biofilm supports cell growth and protects cells from a variety of environmental stresses (Flemming et al., 2016), playing a crucial role in the maintenance of chronic bacterial infections (Dsouza et al., 2024). While its formation has been documented in pathogenic *Leptospira* in various biotic environments (Ristow et al., 2008; Ackermann et al., 2021) and in natural reservoirs (Santos et al., 2021), it is not clear whether it is formed in the bovine genital environment. Nonetheless, the presence of the *wca* family genes strongly suggests this as it is associated with biofilm formation in other Gram-negative bacteria (Yin et al., 2018; Zheng, 2018; Pal et al., 2019). It was demonstrated in *Klebsiella pneumoniae* that *wcaJ* gene encodes a glycosyltransferase that is crucial for the initiation of colanic acid synthesis, a polysaccharide component of the bacterial extracellular matrix. This enzyme facilitates the loading of the first sugar, glucose-1-phosphate, onto the lipid carrier undecaprenyl phosphate, which is a critical step in the biosynthesis of colanic acid (Pal et al., 2019). Although there is no direct evidence of *wca* genes in *Leptospira*, other genomic analyses reveal the presence of analogous genes potentially related to biofilm formation, such as capsular polysaccharide biosynthesis genes and extracellular matrix assembly and transport genes (Davignon et al., 2023). Thibeaux and colleagues (Thibeaux et al., 2020) demonstrated that biofilm production in *L. interrogans* is regulated by intracellular levels of bis-(3′-5′)-cyclic dimeric guanosine monophosphate (c-di-GMP), which underpins the bacterium’s ability to withstand a wide variety of simulated environmental stresses. It is important to note that biofilm formation is associated with a significant increase in antibiotic resistance, which can complicate the treatment of leptospiral infections (Kumar et al., 2016).

Signal transduction in *Leptospira* also plays an important role in adaptation and immune evasion. It has been showed that genes involved in signal transduction mechanisms are abundant in the accessory parts of the *Leptospira* pangenome, suggesting their importance in adaptation and survival in different environments (Lata et al., 2022). These mechanisms may help the bacterium respond rapidly to changes in the host environment, facilitating colonization and evasion of the immune system. In this context, chemotaxis is crucial for the survival of bacteria (Yang and Briegel, 2020). As genital strains of *Leptospira* move toward locations that are considerably more hostile than renal sites, the bacteria must constantly sense its surroundings, respond to different nutrient gradients, and coexist with a greater number of competitors. Consequently, it can be argued that the group of genes associated with signal transduction mechanisms plays a major role in the adaptability of genital strains in the reproductive tract.

The mobilome, defined as all the mobile genetic elements (MGEs) of the microbiome, seems to be another important factor for survival and adaptation of leptospires in the reproductive tract. It has been previously demonstrated that LPS-associated genes is frequent, which can promote the survival of recombinant strains and contribute to antigenic diversity, hindering the host immune response and the development of effective vaccines (Mejía et al., 2022). Moreover, the mobilome influences the composition of microbial communities and the spread of antimicrobial resistance genes and virulence factors via horizontal gene transfer (Carr et al., 2021). Triple the number of genes related to mobilomes, including phages, plasmids, and transposons, was found in genital strains compared with urinary strains (Figure 5). This is unsurprising since the vaginal environment presents greater microbial diversity that facilitates bacterial communication and the exchange of genetic elements. This inter-microbial exchange of genetic elements, including resistance genes, may provide a plausible explanation for the inefficiency of the traditional therapeutic scheme for bovine leptospirosis in eliminating infection in the genital tract. On the other hand, we need to consider that the function of these MGEs in the pathogen has not yet been fully demonstrated, but insights into mobilome-associated genes may inform strategies for limiting genetic exchange that contributes to virulence and resistance.

The present study provides important and impactful initial results based on *in silico* genomic analyses of *L. santarosai*. It is important to note that *in vivo* and *in vitro* studies are also needed to deeply understand how the host-pathogen interaction may influence gene expression by the strains. CRISPR interference (CRISPRi) technology can be used to silence specific genes in L*eptospira*, as demonstrated in the investigation of the role of proteins such as LipL32, LipL41, LipL21 and OmpL1 (Fernandes et al., 2023). The creation of knockdown mutants allows the evaluation of the impact of these genes on virulence and host interaction, using animal models such as the hamster to observe changes in pathogenicity and colonization (Fernandes et al., 2022). Characterization of membrane proteins can be performed to understand their role in adhesion to host tissues and complement evasion, as well demonstrated for TolC Efflux Protein (Hota and Kumar, 2024). Binding and inhibition experiments with specific antibodies can help elucidate functions. Experiments that assess the interaction of *Leptospira* mutants with host molecules, such as extracellular matrix components and component factors, may help identify mechanisms of adhesion and immune evasion, as demonstrate by Zhu et al. (2023).

Future analyses, including that of further genomes from other pathogenic *Leptospira* species from the Sejroe serogroup, such as *L. interrogans* and *L. borgpetersenii*, are necessary to determine whether these results can be replicated in other species with adaptability to the genital site. Transcriptomics studies are also essential to obtain a more comprehensive view of the genes being expressed in the reproductive tract to better elucidate pathogen-host interactions and the mechanisms of pathogenicity by genital strains. Furthermore, targeted and random mutagenesis techniques achieved through the insertion of transposons and genetic complementation of bacteria transformed by synthetic plasmids will enable the exploration of the individual roles of genes. Finally, *in vitro* tests, such as those exploring interaction with defense cells, behavior when exposed to the complement system, and biofilm formation evaluation, and *in vivo* studies, such as those analyzing the effects of mutant strains in the animal model, will be of great importance to this area of study and will be pursued in the future by our scientific team.

Based on comparative genomics analyses, we provide new insights into the pathophysiology of bovine genital leptospirosis, elucidating the evolution of virulence in *Leptospira santarosai* in the genital tract of cows, which, until now, was unknown. Importantly, we find significant differences both at the phylogenetic level and in the functional analysis of the genome when comparing urinary versus genital strains. These latter strains present a greater diversity of genes, with a higher number of genes observed in relation to adhesion, mobile genetic elements, and signal transduction, showing that these processes and factors are key parts of the pathogenesis of BGL. Our results inform the development of a more precise diagnosis of the syndrome, novel therapy proposals, and, importantly, targeted prevention and control strategies.

## Data Availability

The datasets presented in this study can be found in online repositories. The names of the repositories and accession number(s) can be found in the article and in Supplementary material.

## Footnotes

^1^ https://www.ncbi.nlm.nih.gov/datasets/

^2^ https://bioperl.org/

^3^ https://github.com/moshi4/COGclassifier

^4^ https://biopython.org/

^5^ https://pandas.pydata.org/

^6^ https://scipy.org/

^7^ https://www.statsmodels.org/

^8^ https://www.ebi.ac.uk/interpro/search/sequence/
