# Supplementary material for: Comparative genomics of Leptospira santarosai reveals genomic adaptations in bovine genital strains
Source: Front Microbiol. 2025 Jan 7;15:1517151. doi: 10.3389/fmicb.2024.1517151 (PMC11747425; doi:10.3389/fmicb.2024.1517151)
Supplement: Supplementary file 1 [file Table_1.DOCX]

**Supplementary Table 1.** COG Categories enriched in the genital strains specific (GSS) core genome in comparison urinary strain specific (USS) core genome, with the p-value associated.

| COG Category | Corrected *p*-value |
| --- | --- |
| Cell wall/membrane/envelope biogenesis | 0.001714 |
| Signal transduction mechanisms | 0.029663 |
| Mobilome: prophages, transposons | 0.026264 |
