# Supplementary material for: Comparative genomics of Leptospira santarosai reveals genomic adaptations in bovine genital strains
Source: Front Microbiol. 2025 Jan 7;15:1517151. doi: 10.3389/fmicb.2024.1517151 (PMC11747425; doi:10.3389/fmicb.2024.1517151)
Supplement: Supplementary file 2 [file Table_2.DOCX]

**Supplementary Table 2.** COG Categories enriched in the genital strains specific (GSS) core genomes in comparison to the *L. santarosai* pangenome and Pfam domains identified using InterProScan.

| COG CATEGORY | Roary ID | COG ID | COG Name | Pfam Domains |
| --- | --- | --- | --- | --- |
| Cell wall/membrane/envelope biogenesis | Q4558_RS00885 | COG0739 | nlpD |  |
| Cell wall/membrane/envelope biogenesis | Q4558_RS01220 | COG2385 | spoIID |  |
| Cell wall/membrane/envelope biogenesis | Q4558_RS07640 | COG3757 | acm |  |
| Cell wall/membrane/envelope biogenesis | Q4558_RS12405 | COG0463 | wcaA | Glycosyl transferase family 2 (Pfam: PF00535) |
| Cell wall/membrane/envelope biogenesis | Q4558_RS12410 | COG2089 | spsE | NeuB family (Pfam: PF03102) |
| Cell wall/membrane/envelope biogenesis | Q4558_RS12415 | COG1861 | spsF | Cytidylyltransferase (Pfam: PF02348) |
| Cell wall/membrane/envelope biogenesis | Q4558_RS12430 | COG0399 | wecE | DegT/DnrJ/EryC1/StrS aminotransferase family (Pfam: PF01041) |
| Cell wall/membrane/envelope biogenesis | Q4558_RS12440 | COG1861 | SpsF | Cytidylyltransferase (Pfam: PF02348); Oxidoreductase family, NAD-binding Rossmann fold (Pfam: PF01408) |
| Cell wall/membrane/envelope biogenesis | Q4558_RS12445 | COG0399 | wecE | DegT/DnrJ/EryC1/StrS aminotransferase family (Pfam: PF01041) |
| Cell wall/membrane/envelope biogenesis | Q4558_RS12515 | COG1861 | spsF | Cytidylyltransferase (Pfam: PF02348) |
| Cell wall/membrane/envelope biogenesis | Q4558_RS12535 | COG2089 | spsE | NeuB family (Pfam: PF03102) |
| Cell wall/membrane/envelope biogenesis | Q4558_RS12540 | COG3980 | spsG |  |
| Cell wall/membrane/envelope biogenesis | Q4558_RS12550 | COG0399 | wecE | DegT/DnrJ/EryC1/StrS aminotransferase family (Pfam: PF01041) |
| Cell wall/membrane/envelope biogenesis | group_1975 | COG1212 | kdsB | Cytidylyltransferase (Pfam: PF02348) |
| Cell wall/membrane/envelope biogenesis | Q4558_RS12610 | COG3206 | gumC |  |
| Cell wall/membrane/envelope biogenesis | Q4558_RS12640 | COG0463 | wcaA | Glycosyl transferase family 2 (Pfam: PF00535) |
| Cell wall/membrane/envelope biogenesis | Q4558_RS12660 | COG0438 | rfaB | Glycosyl transferases group 1 (Pfam: PF00534) |
| Cell wall/membrane/envelope biogenesis | Q4558_RS12665 | COG0451 | wcaG | GDP-mannose 4,6 dehydratase (Pfam: PF16363) |
| Cell wall/membrane/envelope biogenesis | Q4558_RS12675 | COG0451 | wcaG | NAD dependent epimerase/dehydratase family (Pfam: PF01370) |
| Cell wall/membrane/envelope biogenesis | Q4558_RS12680 | COG1091 | rfbD | RmlD substrate binding domain (Pfam: PF04321) |
| Cell wall/membrane/envelope biogenesis | Q4558_RS12685 | COG1086 | flaA1 | Polysaccharide biosynthesis protein (Pfam: PF02719); Polysaccharide biosynthesis protein C-terminal (Pfam: PF08485) |
| Cell wall/membrane/envelope biogenesis | wecB | COG0381 | wecB | UDP-N-acetylglucosamine 2-epimerase (Pfam: PF02350) |
| Cell wall/membrane/envelope biogenesis | Q4558_RS12695 | COG0438 | rfaB |  |
| Cell wall/membrane/envelope biogenesis | Q4558_RS12700 | COG2148 | wcaJ | Bacterial sugar transferase (Pfam: PF02397) |
| Mobilome: prophages, transposons | Q4558_RS07580 | COG3668 | parE |  |
| Mobilome: prophages, transposons | Q4558_RS11635 | COG2963 | insE |  |
| Mobilome: prophages, transposons | Q4558_RS01005 | COG3299 | jayE | Baseplate J-like protein (Pfam: PF04865) |
| Mobilome: prophages, transposons | Q4558_RS00970 | COG3941 | HI1514 |  |
| Mobilome: prophages, transposons | Q4558_RS00870 | COG4396 | gam | Bacteriophage Mu Gam like protein (Pfam: PF07352) |
| Mobilome: prophages, transposons | Q4558_RS00840 | COG5525 | gpA1 | Phage terminase large subunit gpA, ATPase domain (Pfam: PF05876) |
| Mobilome: prophages, transposons | Q4558_RS00830 | COG2369 |  | Phage Mu protein F like protein (Pfam: PF04233) |
| Mobilome: prophages, transposons | Q4558_RS17820 | COG2801 | tra5 |  |
| Mobilome: prophages, transposons | Q4558_RS16435 | COG4679 |  |  |
| Mobilome: prophages, transposons | Q4558_RS15065 | COG4675 | mdpB |  |
| Mobilome: prophages, transposons | Q4558_RS14970 | COG5362 |  | Terminase large subunit, T4likevirus-type, N-terminal (Pfam: PF03237) |
| Mobilome: prophages, transposons | Q4558_RS16950 | COG3547 |  | Transposase (Pfam: PF01548); Transposase IS116/IS110/IS902 family (Pfam: PF02371) |
| Mobilome: prophages, transposons | Q4558_RS12490 | COG2801 | tra5 | Integrase core domain (Pfam: PF13683) |
| Mobilome: prophages, transposons | Q4558_RS17905 | COG3328 | IS285 | Transposase, Mutator family (Pfam: PF00872) |
| Mobilome: prophages, transposons | Q4558_RS12710 | COG3039 | IS5 | Transposase domain (DUF772) (Pfam: PF05598); Transposase DDE domain (Pfam: PF01609) |
| Mobilome: prophages, transposons | Q4558_RS14590 | COG5004 | gpX |  |
| Mobilome: prophages, transposons | Q4558_RS14565 | COG3299 | jayE | Baseplate J-like protein (Pfam: PF04865) |
| Mobilome: prophages, transposons | Q4558_RS17900 | COG2963 | insE | Transposase (Pfam: PF01527) |
| Signal transduction mechanisms | Q4558_RS00465 | COG0840 | tar | Methyl-accepting chemotaxis protein (MCP) signalling domain (Pfam: PF00015) |
| Signal transduction mechanisms | creC | COG0642 | baeS | Histidine kinase-, DNA gyrase B-, and HSP90-like ATPase (Pfam: PF02518) |
| Signal transduction mechanisms | Q4558_RS12965 | COG0666 | ANKYR | Ankyrin repeats (3 copies) (Pfam: PF12796) |
| Signal transduction mechanisms | Q4558_RS14025 | COG0664 | crp | Cyclic nucleotide-binding domain (Pfam: PF00027); Ion transport protein (Pfam: PF00520) |
| Signal transduction mechanisms | Q4558_RS03980 | COG0666 | ANKYR |  |
| Signal transduction mechanisms | Q4558_RS16090 | COG0840 | tar | Methyl-accepting chemotaxis protein (MCP) signalling domain (Pfam: PF00015) |
| Signal transduction mechanisms | Q4558_RS07135 | COG0666 | ANKYR | Ankyrin repeats (3 copies) (Pfam: PF12796); Ankyrin repeats (3 copies) (Pfam: PF12796) |
| Signal transduction mechanisms | Q4558_RS03160 | COG1366 | spoIIAA | STAS domain (Pfam: PF01740) |
| Signal transduction mechanisms | Q4558_RS03695 | COG4191 |  | Histidine kinase-, DNA gyrase B-, and HSP90-like ATPase (Pfam: PF02518); Protein of unknown function (DUF3365) (Pfam: PF11845) |
| Signal transduction mechanisms | Q4558_RS03700 | COG2114 | acyC | Adenylate and Guanylate cyclase catalytic domain (Pfam: PF00211); Family of unknown function (DUF5939) (Pfam: PF19363) |
| Signal transduction mechanisms | Q4558_RS03715 | COG0631 | PTC1 |  |
| Signal transduction mechanisms | Q4558_RS05625 | COG0666 | ANKYR | Ankyrin repeats (3 copies) (Pfam: PF12796); Ankyrin repeats (3 copies) (Pfam: PF12796) |
| Signal transduction mechanisms | Q4558_RS04140 | COG3920 |  | Histidine kinase (Pfam: PF07568) |
