# Supplementary material for: Comparative genomics of Leptospira santarosai reveals genomic adaptations in bovine genital strains
Source: Front Microbiol. 2025 Jan 7;15:1517151. doi: 10.3389/fmicb.2024.1517151 (PMC11747425; doi:10.3389/fmicb.2024.1517151)
Supplement: Supplementary file 3 [file Table_3.DOCX]

**Supplementary Table 3.** Percent of genital (Table A) and urinary (Table B) genes orthologues.

**Table A**

| **Accession** | **Strain** | **Serovar** | **Percent of genital genes orthologues** |
| --- | --- | --- | --- |
| GCF_001952685.1 | M4/98 | Guaricura | 71,88% |
| GCF_030546545.1 | U76 |  | 68,17% |
| GCF_000343395.1 | Oregon | Szwajizak | 68,17% |
| GCF_001569335.1 | 56663 |  | 68,17% |
| GCF_001569265.1 | 56163 |  | 67,90% |
| GCF_000244555.1 | CBC379 |  | 67,11% |
| GCF_000244735.1 | MOR084 |  | 66,58% |
| GCF_000348015.1 | CBC1531 |  | 66,05% |
| GCF_001981505.1 | M52/8-19 |  | 64,19% |
| GCF_000244475.1 | 200702252 |  | 64,19% |
| GCF_001008335.2 | U164 |  | 63,93% |
| GCF_000306575.1 | 200403458 |  | 63,93% |
| GCF_030023785.1 | CR0421 |  | 62,86% |
| GCF_030023815.1 | LSU1013 |  | 62,60% |
| GCF_000244655.1 | HAI1380 |  | 61,80% |
| GCF_000243835.1 | MAVJ 401 | Arenal | 61,80% |
| GCF_001584305.1 | C216 |  | 59,15% |
| GCF_030023865.1 | CR2020 |  | 57,82% |
| GCF_000346915.1 | HAI1349 |  | 54,11% |
| GCF_001981425.1 | 2ACAP | Bananal | 51,99% |
| GCF_001952675.1 | LO-9 | Grippotyphosa | 51,19% |
| GCF_001981455.1 | M72/6-6 | Grippotyphosa | 50,13% |
| GCF_000306455.1 | AIM |  | 48,01% |
| GCF_001569285.1 | 56164 |  | 47,21% |
| GCF_001568365.1 | 56198 |  | 46,95% |
| GCF_030023985.1 | CR0521 |  | 46,68% |
| GCF_001008325.2 | U233 |  | 46,68% |
| GCF_000217455.2 | 2000030832 |  | 46,42% |
| GCF_000332395.2 | 1342KT | Shremani | 46,42% |
| GCF_026914325.1 | DCP-017 |  | 46,42% |
| GCF_000313175.2 | LT 821 | Shermani | 46,42% |
| GCF_030024155.1 | CR2921 |  | 46,15% |
| GCF_000244575.1 | CBC523 |  | 46,15% |
| GCF_030023905.1 | 202101808 |  | 45,36% |
| GCF_001569305.1 | 56274 |  | 45,36% |
| GCF_000246375.1 | 7 | Arenal | 40,32% |
| GCF_030023665.1 | Rr5 |  | 39,52% |
| GCF_000246395.1 | 11 | Arenal | 38,99% |
| GCF_000244675.1 | HAI821 |  | 38,20% |
| GCF_000244615.1 | HAI134 |  | 38,20% |
| GCF_000306475.1 | JET |  | 37,93% |
| GCF_030024005.1 | CR0821 |  | 37,14% |
| GCF_030023595.1 | CR2821 |  | 36,87% |
| GCF_030023745.1 | inciensa04 |  | 36,87% |
| GCF_000244795.1 | ZUN179 |  | 36,34% |
| GCF_000332435.1 | ST188 |  | 36,07% |
| GCF_001569295.1 | 56215 |  | 35,81% |
| GCF_000216275.1 | 2000027870 |  | 35,54% |
| GCF_030023615.1 | CR1421 |  | 35,28% |
| GCF_030023805.1 | CR2120 |  | 35,28% |
| GCF_030023765.1 | MMD3 |  | 33,95% |
| GCF_030023725.1 | JICH05 |  | 33,69% |
| GCF_030023925.1 | CR1821 |  | 33,69% |
| GCF_001568375.1 | 56180 |  | 33,69% |
| GCF_030023695.1 | CR2621 |  | 33,16% |

**Table B**

| **Accession** | **Strain** | **Serovar** | **Percent of urinary genes orthologues** |
| --- | --- | --- | --- |
| GCF_001981455.1 | M72/6-6 | Grippotyphosa | 95,18% |
| GCF_001981505.1 | M52/8-19 |  | 95,18% |
| GCF_000244475.1 | 200702252 |  | 90,36% |
| GCF_000306575.1 | 200403458 |  | 90,36% |
| GCF_030023665.1 | Rr5 |  | 89,16% |
| GCF_001952675.1 | LO-9 | Grippotyphosa | 89,16% |
| GCF_001981425.1 | 2ACAP | Bananal | 89,16% |
| GCF_026914325.1 | DCP-017 |  | 87,95% |
| GCF_000306455.1 | AIM |  | 86,75% |
| GCF_000244655.1 | HAI1380 |  | 84,34% |
| GCF_000313175.2 | LT 821 | Shermani | 84,34% |
| GCF_000244575.1 | CBC523 |  | 83,13% |
| GCF_000244555.1 | CBC379 |  | 83,13% |
| GCF_000332395.2 | 1342KT | Shremani | 83,13% |
| GCF_001952685.1 | M4/98 | Guaricura | 83,13% |
| GCF_000244795.1 | ZUN179 |  | 81,93% |
| GCF_030024155.1 | CR2921 |  | 80,72% |
| GCF_030023905.1 | 202101808 |  | 80,72% |
| GCF_000244675.1 | HAI821 |  | 79,52% |
| GCF_030023925.1 | CR1821 |  | 77,11% |
| GCF_000306475.1 | JET |  | 77,11% |
| GCF_030023725.1 | JICH05 |  | 74,70% |
| GCF_000332435.1 | ST188 |  | 73,49% |
| GCF_001569295.1 | 56215 |  | 72,29% |
| GCF_000244735.1 | MOR084 |  | 72,29% |
| GCF_030023785.1 | CR0421 |  | 69,88% |
| GCF_030023765.1 | MMD3 |  | 66,27% |
| GCF_000216275.1 | 2000027870 |  | 65,06% |
| GCF_000348015.1 | CBC1531 |  | 65,06% |
| GCF_000246395.1 | 11 | Arenal | 63,86% |
| GCF_030023615.1 | CR1421 |  | 63,86% |
| GCF_030024005.1 | CR0821 |  | 62,65% |
| GCF_030023695.1 | CR2621 |  | 62,65% |
| GCF_030023595.1 | CR2821 |  | 62,65% |
| GCF_030023805.1 | CR2120 |  | 62,65% |
| GCF_000244615.1 | HAI134 |  | 61,45% |
| GCF_030023745.1 | inciensa04 |  | 61,45% |
| GCF_000243835.1 | MAVJ 401 | Arenal | 61,45% |
| GCF_001568375.1 | 56180 |  | 61,45% |
| GCF_030023985.1 | CR0521 |  | 60,24% |
| GCF_001569305.1 | 56274 |  | 60,24% |
| GCF_001584305.1 | C216 |  | 59,04% |
| GCF_000217455.2 | 2000030832 |  | 59,04% |
| GCF_030023865.1 | CR2020 |  | 59,04% |
| GCF_000246375.1 | 7 | Arenal | 57,83% |
| GCF_000346915.1 | HAI1349 |  | 57,83% |
| GCF_001568365.1 | 56198 |  | 57,83% |
| GCF_030546565.1 | 2014_VF66 |  | 57,83% |
| GCF_030023815.1 | LSU1013 |  | 55,42% |
| GCF_001569335.1 | 56663 |  | 55,42% |
| GCF_001569285.1 | 56164 |  | 54,22% |
| GCF_000343395.1 | Oregon | Szwajizak | 54,22% |
| GCF_030546555.1 | VF237 |  | 53,01% |
| GCF_030023685.1 | 2015VF237 |  | 53,01% |
| GCF_001569265.1 | 56163 |  | 53,01% |
| GCF_030023605.1 | 2013VF52 |  | 51,81% |
| GCF_030546585.1 | 2013_VF52 |  | 51,81% |
